# Effects of Tween 80 on Growth and Biofilm Formation in Laboratory Media

**DOI:** 10.3389/fmicb.2016.01878

**Published:** 2016-11-22

**Authors:** Christina K. Nielsen, Jørgen Kjems, Tina Mygind, Torben Snabe, Rikke L. Meyer

**Affiliations:** ^1^Interdisciplinary Nanoscience Center, Aarhus UniversityAarhus, Denmark; ^2^DuPont Nutrition BiosciencesBrabrand, Denmark

**Keywords:** Tween 80, polysorbate 80, biofilm, isoeugenol, emulsifier, *Staphylococcus aureus*

## Abstract

Tween 80 is a widely used non-ionic emulsifier that is added to cosmetics, pharmaceuticals, and foods. Because of its widespread use we need to understand how it affects bacteria on our skin, in our gut, and in food products. The aim of this study is to investigate how Tween 80 affects the growth and antimicrobial susceptibility of *Staphylococcus aureus*, *Listeria monocytogenes*, and *Pseudomonas fluorescens*, which are common causes of spoilage and foodborne illnesses. Addition of 0.1% Tween 80 to laboratory growth media increased the growth rate of planktonic *S. aureus* batch cultures, and it also increased the total biomass when *S. aureus* was grown as biofilms. In contrast, Tween 80 had no effect on batch cultures of *L. monocytogenes*, it slowed the growth rate of *P. fluorescens*, and it led to formation of less biofilm by both *L. monocytogenes* and *P. fluorescens.* Furthermore, Tween 80 lowered the antibacterial efficacy of two hydrophobic antimicrobials: rifampicin and the essential oil isoeugenol. Our findings underline the importance of documenting indirect effects of emulsifiers when studying the efficacy of hydrophobic antimicrobials that are dispersed in solution by emulsification, or when antimicrobials are applied in food matrixes that include emulsifiers. Furthermore, the species-specific effects on microbial growth suggests that Tween 80 in cosmetics and food products could affect the composition of skin and gut microbiota, and the effect of emulsifiers on the human microbiome should therefore be explored to uncover potential health effects.

## Introduction

Tween 80 (polysorbate 80, polyoxyethylene sorbitan monooleate) is a non-ionic surfactant that is widely used as an emulsifier in cosmetics, pharmaceuticals and food products. It is approved by the US Food and Drug Administration for use in up to 1% in selected foods (Chassaing et al., 2015). Tween 80 has attracted some attention in recent years because studies have shown that addition of 1% Tween 80 to drinking water leads to inflammation and increased adiposity in mice (Chassaing et al., 2015). The effects were not seen in germ-free mice and were therefore ascribed to changes in the gut microbiota after intake of Tween 80. With the increasing awareness of correlations between gut microbiota and human health, it is of great importance to understand how food additives affect the human microbiome.

*Staphylococcus aureus* is a Gram-positive pathogen that causes many acute and chronic infections. It is commonly associated with foodborne illnesses (Seow et al., 2014) and it is responsible for many instances of abscesses, septicemia, arthritis, and endocarditis (Kenny et al., 2009). Therefore, extensive research has been undertaken to understand the factors that affect the life cycle and virulence of *S. aureus. Listeria monocytogenes* is likewise a Gram-positive pathogen associated with foodborne illnesses (Listeriosis) (Pan et al., 2014), whereas *Pseudomonas fluorescens* is a Gram-negative bacterium associated with food spoilage (Rajmohan et al., 2002).

We hypothesize that Tween 80 affects bacterial growth by directly affecting (1) viability and growth rates, and (2) biofilm formation. Earlier studies have shown that addition of Tween 80 reduced bacterial adhesion and inhibited biofilm formation of *Pseudomonas* sp. *AKS2* (Tribedi and Sil, 2013) and several isolates of *Pseudomonas aeruginosa, Escherichia coli, S. aureus, Staphylococcus epidermidis*, and more (Toutain-Kidd et al., 2009). However, in all cases, Tween 80 was added before the attachment phase of biofilm development, and therefore reflects its effect on bacterial attachment to abiotic surfaces. This study adds to existing knowledge by investigating how Tween 80 affects planktonic growth and biofilm development of *S. aureus*, *L. monocytogenes*, and *P. fluorescens*, adding Tween 80 at different stages of biofilm development. Tween 80 may affect food pathogens directly, or it may affect food safety in general by altering the efficiency of antimicrobials added as preservatives. We therefore also investigated if Tween 80 affected the antimicrobial potency of two hydrophobic antimicrobial compounds.

## Materials and Methods

### Preparation of Bacterial Cultures

*Staphylococcus aureus* (DSM20231) and *L. monocytogenes* (NCTC 12426) were purchased from the German culture collection (DSMZ), and *P. fluorescens* AH2 (Gram et al., 1990) was kindly donated by Professor Lone Gram at the Danish Technical University. Cultures were stored in 30% glycerol at -80°C, and streaked on to tryptic soy agar. Overnight cultures were prepared by inoculating from one colony to a 100 mL Erlenmeyer flask containing 20 mL Tryptic Soy Broth (TSB; 30 g/L pH adjusted to 6 with HCl, Sigma-Aldrich) and incubated overnight at 25°C with shaking at 180 rpm. Three biological replica inoculated from individual colonies were prepared for each experiment.

### Effect of Tween 80 on Planktonic Growth

Overnight cultures of *S. aureus, L. monocytogenes*, or *P. fluorescens* were prepared as described and diluted 1000 times in either TSB or TSB with 0.1% (v/v) Tween 80 in 96-well plates. Growth curves were recorded by measuring the optical density at 600 nm (OD_600_) every 20 min during incubation at 25°C (BioTek, PowerWave XS2, Holm&Halby).

### Effect of Tween 80 on Biofilm Development

Overnight cultures of *S. aureus, L. monocytogenes*, or *P. fluorescens* were prepared as described and 160 μL was transferred to wells in a 96-well plate. A peg lid (Nunc^TM^ Immuno^TM^ TSP lid, product no 445497, Thermo Scientific) was added and left for 10 min at room temperature to inoculate the pegs. The peg lid was then transferred to a new plate with 160 μL/well TSB with or without Tween 80 (at 1.0, 0.5, or 0.1% (v/v)) and incubated at 25°C with shaking at 50 rpm. Transfer of the peg lid to TSB with Tween 80 was either done immediately after inoculation, or after biofilms had been allowed to develop for 24 or 48 h.

At the end of the incubation, the biofilm biomass was quantified by crystal violet staining. The peg lid was washed twice by insertion into microtiter plates with 180 μL/well of phosphate buffered saline (PBS), and then incubated for 5 min in 0.5% (v/v) Gram’s crystal violet solution (Sigma-Aldrich) freshly prepared in demineralized water. The peg lid was then washed twice by insertion into in plates with 180 μL/well of demineralized water, and crystal violet was then extracted from the biofilm by incubation for for 5 min in a plate with 180 μL/well 96% ethanol. Finally the optical density the extracted crystal violet was measured at 585 nm. Three biological replicas and three technical replicas were prepared for each experiment.

More detailed measurements were performed for all three bacterial strains to observe the biomass development with 12 h intervals in the presence or absence of 0.1% (v/v) Tween 80 or a mix of 0.1% (v/v) oleic acid and 0.1% (v/v) Tween 80. Biofilms were grown in TSB for either 24 or 48 h as described above and then transferred to a microtiter plate with TSB or TSB with 0.1% (v/v) Tween 80, or 0.1% (v/v) oleic acid plus 0.1% (v/v) Tween 80. At 12 h intervals, incubations were stopped and the biofilm biomass was quantified by crystal violet staining as described above.

### Morphology of Tween 80-treated *S. aureus* Biofilms

Overnight cultures of *S. aureus* were prepared as described above, and 100 μL/well were added to 96-well plates suitable for microscopy (Ibidi, Germany, hydrophobic, uncoated). After 30 min, the bacterial suspension was carefully replaced with 200 μL TSB or TSB with 0.1% (v/v) Tween 80, and the plate was incubated at 25°C for 48 h. The liquid was removed by aspiration and the wells were washed twice with PBS. Finally, the PBS was replaced with 200 μL TSB or TSB with 0.1% (v/v) Tween 80, and the plate was incubated at 25°C for additional 24 h. Next, the liquid above the biofilm was carefully replaced with PBS, ensuring that the biofilm did not dry out in the process. Finally, the DNA-binding stains TOTO-1 (Life Technologies) and SYTO 60 (Life Technologies) were added from stock solutions to obtain working concentrations of 2 and 10 μM, respectively. A LSM700 confocal laser scanning microscope (Zeiss) was used to acquire images with a 40× (NA 1.3) oil immersion objective, and the software Zen was used for image processing. Quantitative image processing was done with ImageJ using the plugin Comstat 2.1.

### Effect of Tween 80 on Minimum Biofilm Eradication Concentration of Rifampicin and Isoeugenol

To investigate whether Tween 80 could change the antimicrobial activity of the two hydrophobic antimicrobials rifampicin and isoeugenol, we determined the minimum biofilm eradication concentration (MBEC) in the presence and absence of 0.1% (v/v) Tween 80. Overnight cultures of *S. aureus* were transferred a 96-well plate (160 μl/well) and a peg lid was inserted for 10 min at room temperature to inoculate the pegs. The peg lid was then transferred to a new 96-well plate with 160 μL/well TSB and incubated at 25°C with orbital shaking at 180 rpm for 48 h. Next, the peg lid was washed twice by insertion into 96-well plates with 180 μL/well PBS and finally transferred to a new plate containing a dilution series of either rifampicin (Sigma-Aldrich) or isoeugenol (Sigma-Aldrich) in TSB or TSB with 0.1% (v/v) Tween 80. The dilution series was made by preparing a stock solution of 128 mg/L of rifampicin or 12000 mg/L of isoeugenol in TSB or TSB with 0.1% (v/v) Tween 80. This solution was serially diluted with 2/3 step sizes in either TSB or TSB with 0.1% (v/v) Tween 80. The stock solution was used as the first concentration in the dilution series. The peg lid was incubated in the plate with antimicrobials at 25°C with 180 rpm for 24 h before washing by into 96-well plates with 180 μL/well PBS. The peg lid was finally transferred to a plate with 180 μL/well TSB and incubated for 72 h at 25°C with 180 rpm shaking. If any bacteria in the biofilms survived the antimicrobial treatment, it would result in planktonic growth in the wells of this “recovery plate.” OD_620_ was measured in the recovery plate just before and after the 72 h incubation, and the MBEC was defined as the lowest concentration in no detectable growth in the recovery plate.

### Particle Size Distribution

To understand whether Tween 80 could affect nutrient availability in TSB, the particle size distribution of TSB and TSB with Tween 80 was measured by Dynamic Light Scattering (DLS). DLS is a technique that uses the fluctuations in scattered laser light to calculate a hydrodynamic radius of particles in suspension. Samples were transferred to clear disposable zeta cells and mounted in a Nano-ZS (Malvern Instruments). Triplicate measurements of each sample were recorded and results are presented as the average of these. We performed the measurements at 25°C with an equilibration time of 60 s. Sizes are given as the *Z*-average value.

### Permeabilization of Model Membrane

In order to study if Tween 80 could cause membrane permeabilization, a model membrane system was prepared as calcein-loaded vesicles consisting of 50% cardiolipin (Sigma-Aldrich) and 50% phosphatidylglycerol (Avanti Polar Lipids). A thin film was made by mixing 5 mg cardiolipin and 5 mg phosphatidylglycerol in 300 μL chloroform in a glass vessel. The solution was shaken until the solids were dissolved. Next, chloroform was evaporated with nitrogen gas creating a thin film on the walls of the glass vessel. A 70 mM calcein (Fluka) stock solution was prepared in TSB, and 1 mL was transferred to the glass vessel with the thin film and vortexed thoroughly. The content was then immersed into liquid nitrogen (freezing) and thawed again in warm water twelve times. In order to obtain membrane vesicles with uniform size, the solution was extruded 12 times through a 0.2 μm filter. Finally, the solution was run through a PD-10 desalting column (GE Healthcare) equilibrated with TSB to separate membrane-enclosed calcein from free calcein. The fraction containing membrane-enclosed calcein was collected from the column and stored at 4°C for the next day, where it was diluted 75 times and transferred to a microtiter plate (200 μL/well).

Calcein leakage was measured as an increase in fluorescence intensity, as the fluorescence of calcein in the vesicles is initially low due to quenching, and would increase as calcein leaked through the membrane. Fluorescence intensity was measured with VarioSkan Flash fluorometer (Thermo Scientific) at 485 nm excitation and 518 nm emission. The background fluorescence from calcein (*F*_0_) was measured, and Tween 80 was added in final concentrations between 0.05 and 1% (v/v). The fluorescence was recorded for triplicate samples 1 h after addition of Tween 80. Finally, Triton X-100 (Fluka) was added to a final concentration of 0.5% to record the fluorescence intensity when vesicles were fully permeabilized (*F*_max_). Results are given as percentage of calcein release relative to the release obtained with Triton X:

$$release\left(\%\right)=\frac{F-F_{0}}{F_{\max}-F_{0}}\cdot 100\%$$

### Statistics

Data for biofilm development and analysis of confocal images with ImageJ were subjected to Student’s *t*-test by using the software Stata. For all tests, a level of *p* < 0.05 was considered significant.

## Results

### Tween 80 Slowed the Planktonic Growth Rate of *S. aureus* and *P. fluorescens*

**FIGURE 1:**
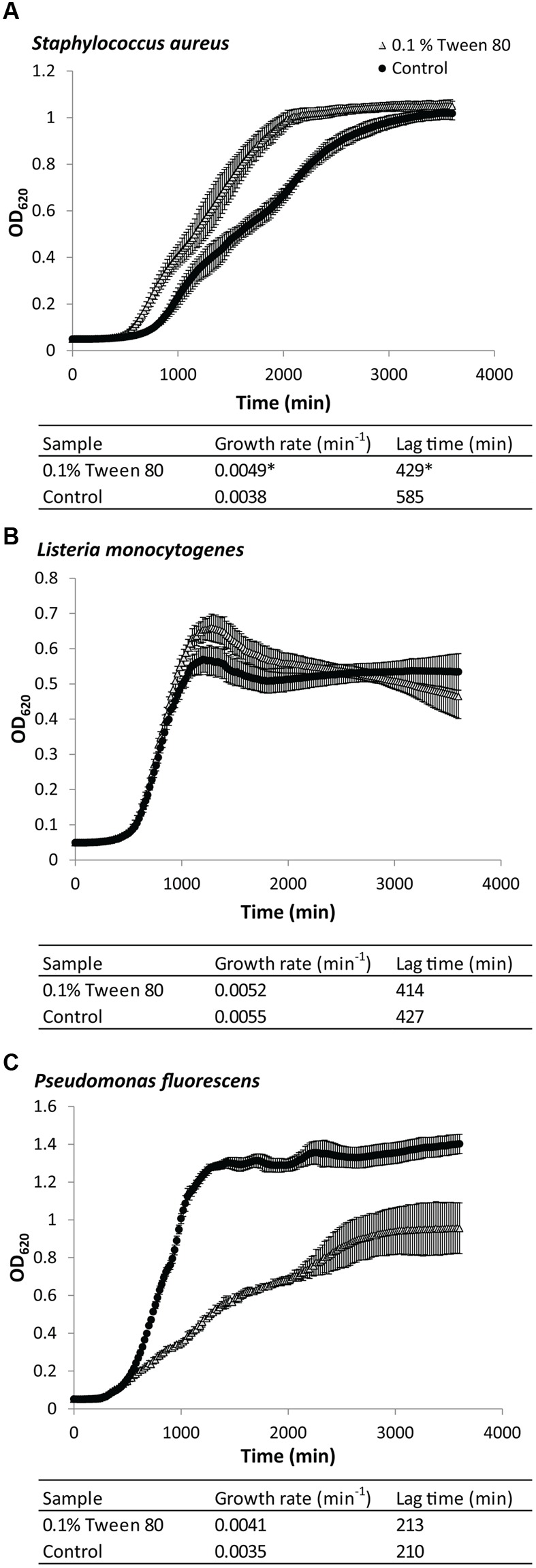
**Growth curves of (A)** *Staphylococcus aureus*, **(B)** *Pseudomonas fluorescens*, or **(C)** *Listeria monocytogenes* grown in either Tryptic Soy Broth (TSB) or TSB with 0.1 (v/v) % Tween 80. Error bars show standard deviation of the mean (*n* = 9). Growth rates and lag times are included. Statistical significance compared to control is marked by ^∗^(*p* < 0.05).

Addition of 0.1% (v/v) Tween 80 affected planktonic growth of *S. aureus, L. monocytogenes*, and *P. fluorescens* in very different ways (**Figure 1**). Growth with Tween 80 increased the growth rate for planktonic *S. aureus* (**Figure 1A**). The lag phase also appeared to be shorter, but this could simply appear to be the case due to the faster growth rate, resulting in these cultures arriving faster at the cell density required for spectrophotometric detection. *L. monocytogenes* was unaffected by addition of Tween 80 to the media (**Figure 1B**). The growth rate of *P. fluorescens* was initially higher than in cultures grown with Tween 80, but it then decreased again, and the maximum OD was substantially lower than the control (**Figure 1C**).

### Effect of Tween 80 on Biofilm Growth

**FIGURE 2:**
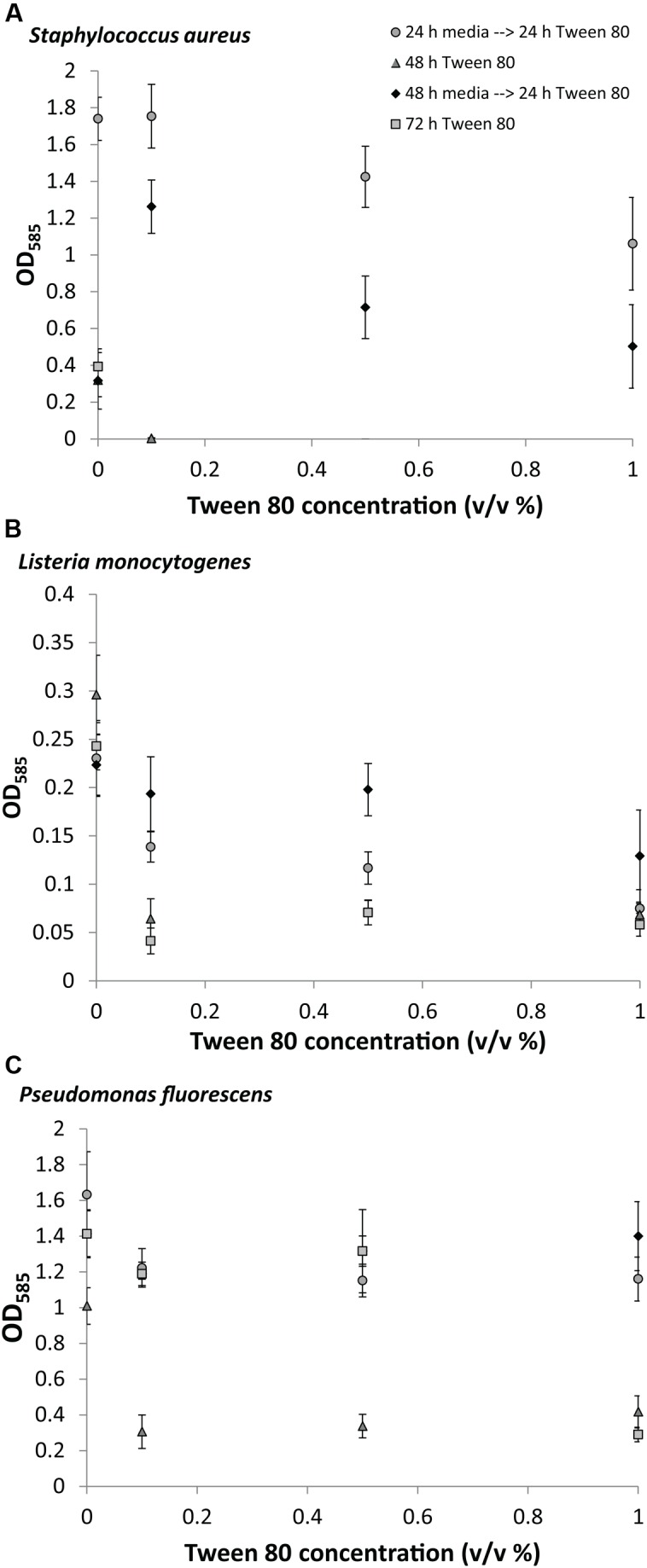
**Crystal violet staining of (A)** *S. aureus*, **(B)** *L. monocytogenes*, **(C)** *P. fluorescens* after growth of biofilms for 0, 24, or 48 h in TSB followed by treatment with 0.1, 0.5, or 1.0% (v/v) Tween 80 for 24, 48, or 72 h. Error bars show standard deviation of the mean. All treatments were statistically significant (*p* < 0.05) from the control, except *S. aureus* 0.1% Tween (24 h→24 h); *L. monocytogenes* 0.1% and 0.5% Tween (48 h→24 h); and *P. fluorescens* 0.5% and 1% (48 h→24 h).

We measured the biomass of *S. aureus, L. monocytogenes*, and *P. fluorescens* biofilms after replacing the media with TSB containing 0.1% (v/v), 0.5% (v/v), or 1.0% (v/v) Tween 80. Tween 80 has a critical micelle concentration of 0.001% at 25°C (Vidal-Paruta and King, 1964), meaning that the surfactant was added at concentrations were micelles would be formed. In the first experiment, Tween 80 was included from the beginning of the 48 or 72 h incubation to determine the effect of Tween 80 on the establishment of biofilms. This resulted in no or very little biofilm formation for all organisms at all concentrations of Tween 80 (**Figure 2**). In the second experiment, we let the biofilms grow for either 24 or 48 h in TSB before replacing the media with TSB containing Tween 80, and allowing for 24 h subsequent growth. These experiments would determine the effect of Tween 80 on already established biofilms. Addition of Tween 80 to a 24 h old biofilm had either no effect or impaired further biofilm development for all three organisms. If the biofilm was grown for 48 h before addition of Tween 80, we saw a concentration dependent stimulation of *S. aureus* biofilm formation with the lowest concentration (0.1% (v/v)) resulting in the highest stimulation of biofilm formation. This effect was only seen for *S. aureus*. Addition of Tween 80 to *P. fluorescens* and *L. monocytogenes* had either no effect or reduced the further growth of biofilm.

**FIGURE 3:**
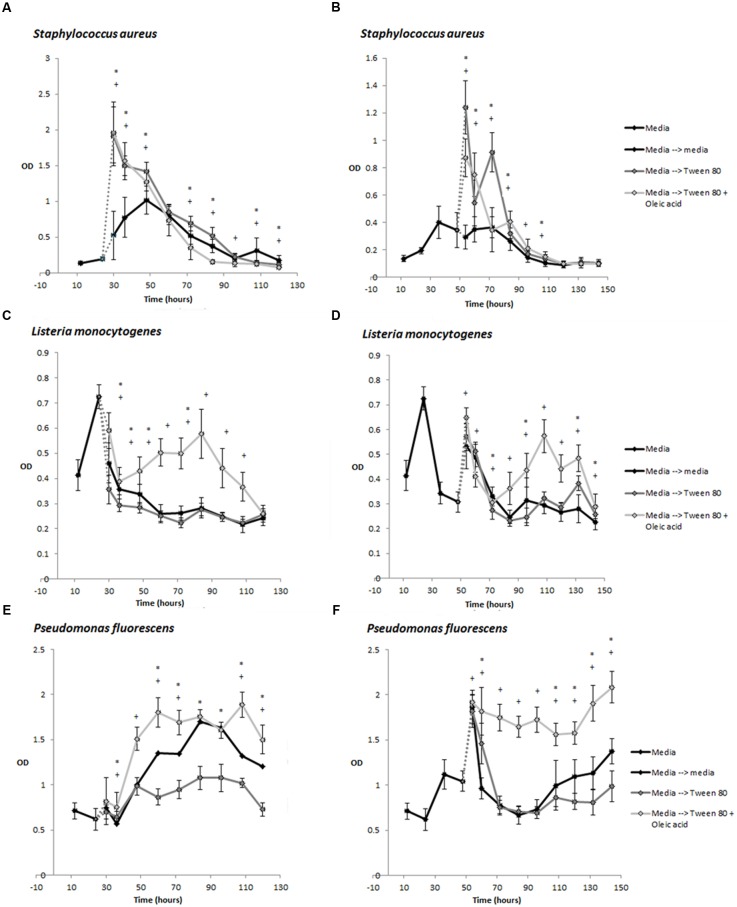
**Development of biomass over time measured by crystal violet staining (OD_585_ measurement)** for *S. aureus* **(A,B)**, *L. monocytogenes* **(C,D)**, and *P. fluorescens* **(E,F)**. Biofilms were grown in TSB for 24 h **(A,C,E)** or 48 h **(B,D,F)** before replacement of the supernatant with TSB containing 0.1% Tween 80 or 0.1% Tween 80 + 0.1% Oleic acid. Error bars show standard deviation of the mean (*n* = 18). Biofilms treated with Tween 80 or Tween 80 + Oleic acid were compared to control biofilms. Statistical significance (*p* < 0.05) is marked by ^∗^ for Tween 80-treated biofilms and + for Tween 80 and Oleic acid-treated samples.

Under some conditions, Tween 80 can undergo hydrolysis at the fatty acid ester bond, releasing oleic acid (Kerwin, 2008). During our studies, we discovered that the commercial Tween 80 solution did contain some oleic acid. Oleic acid has been shown to inhibit *S. aureus* biofilm formation if the fatty acid is present during biofilm formation (Stenz et al., 2008). However, the authors also showed that oleic acid could stimulate *S. aureus* biofilm formation if it was introduced after the initiation of biofilm growth. It was therefore relevant to determine if the effect we observed were caused by Tween 80 or oleic acid. We therefore compared the effect of Tween 80 and oleic acid by quantifying biofilm biomass after replacing the overlying media of 24 or 48 h old biofilms with TSB containing either 0.1% Tween 80, 0.1% oleic acid or both. Tween 80 stimulated the biomass of *S. aureus* biofilms while this did not appear to be the case for oleic acid (**Figures 3A–C**). Addition of Tween 80 to *L. monocytogenes* and *P. fluorescens* biofilms resulted in less biomass at most time points, whereas addition of Tween 80 with oleic acid increased the biomass (**Figures 3C–F**).

### Effect of Tween 80 on Biofilm Morphology

**FIGURE 4:**
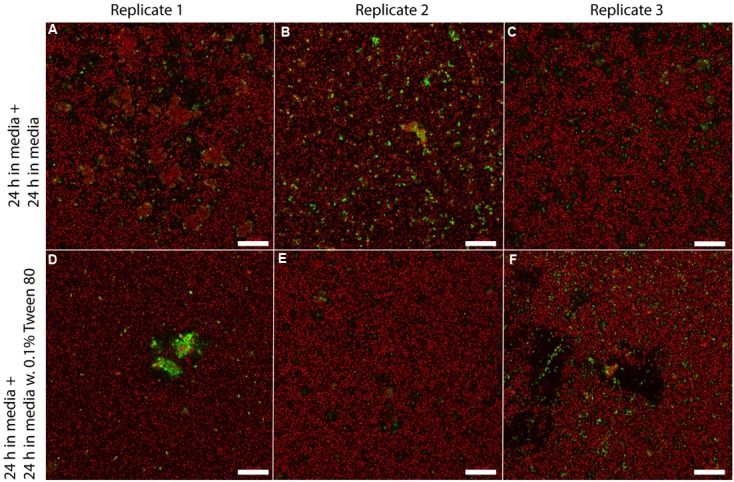
**Confocal laser scanning microscopy images of three replicate *S. aureus* biofilms** grown for 48 h in TSB media and transferred to either fresh media **(A–C)** or 0.1% Tween 80 **(D–F)** followed by growth for 24 h. SYTO 60 (red) stains living bacteria while TOTO-1 (green) stains dead bacteria and extracellular DNA. Scale bar = 20 μm.

**FIGURE 5:**
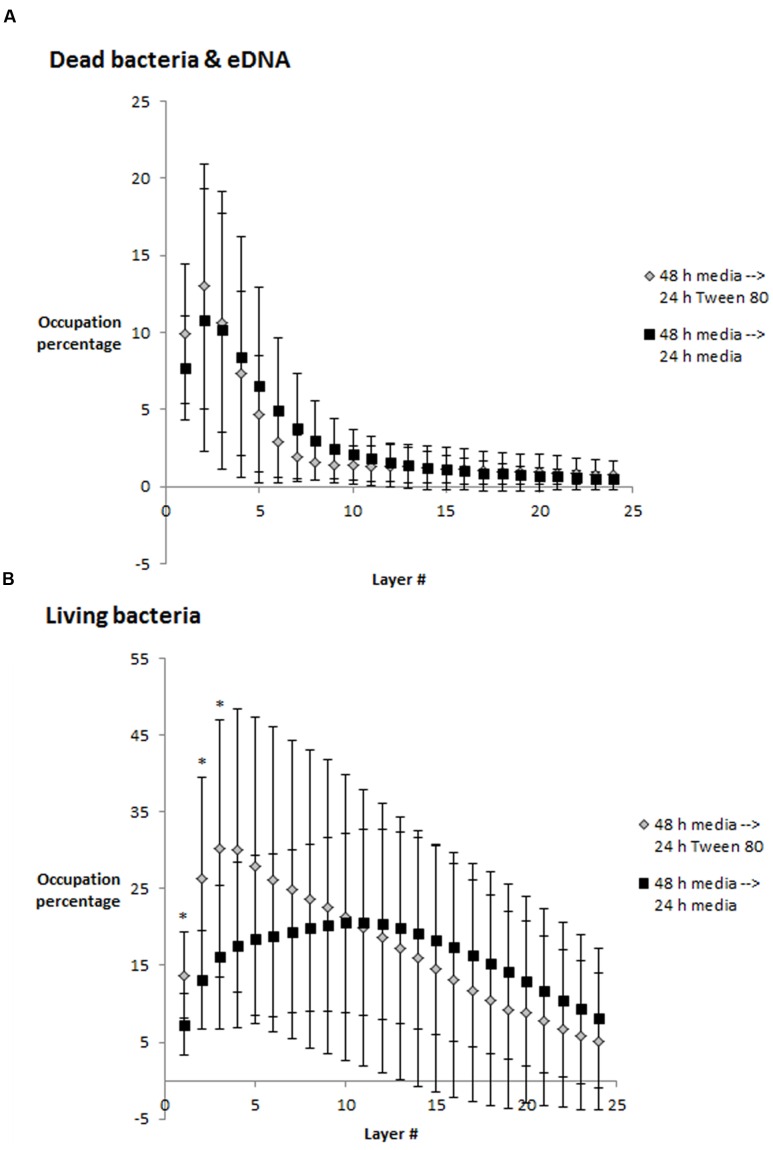
**Occupied area (in percentage) per layer in z-stacks from confocal laser scanning microscopy images. (A)** Dead bacteria and eDNA, **(B)** Living bacteria. Error bars show standard deviation of the mean (*n* = 10). Statistical significance (*p* < 0.05) is marked by ^∗^. Each layer corresponds to 0.53 μm.

We visualized *S. aureus* biofilms by confocal laser scanning microscopy using SYTO 60 and TOTO-1 staining (**Figure 4**). The biofilms were grown in TSB for 48 h, and the media was then replaced with TSB or TSB with 0.1% Tween 80 followed by 24 h incubation before visualizing the biofilm. We did not observe any apparent differences in biofilm morphology. However, there was a higher number of living bacteria per area in the layer of bacteria that was close to the surface in the Tween 80-treated biofilms compared to the control (**Figure 5**). The differences were statistically significant for the three first images in the z-stack, corresponding to 1.6 μm.

### Tween 80 Lowered the Antibacterial Efficacy of Two Hydrophobic Antimicrobials

Since Tween 80 is a surfactant, it can form micelles and emulsions encapsulating hydrophobic substances. We therefore suspected that Tween 80 might influence the antibacterial efficacies of hydrophobic antimicrobials. Essential oils have gained much attention recently for their potential use as natural food preservatives. Since essential oils are hydrophobic, emulsifiers such as Tween 80 are often added to ensure dispersion. We therefore investigated if Tween 80 could affect the antibacterial efficacy of the essential oil isoeugenol, and of the hydrophobic antibiotic rifampicin. The MBEC values of rifampicin and isoeugenol were measured against 48 h old *S. aureus* biofilms with and without addition of 0.1% Tween 80. We observed that addition of Tween 80 to both antimicrobials increased the concentration required to eradicate *S. aureus* biofilms (**Table 1**). The MBEC of Tween 80 alone was included as a control and was higher than the concentrations tested in this study (>100,000 mg/L).

**Table 1:** Minimum Biofilm Eradication Concentrations (MBEC) in mg/L for rifampicin and isoeugenol against *Staphylococcus aureus.*

MBEC [mg/L]	With Tween 80	Without Tween 80
Rifampicin	17	<2
Isoeugenol	6,000	1,500
		
Tween	–	>100,000

*The value for pure Tween 80 was included as a control*.

### Tween 80 Reduced the Sizes of Particles in Media

We hypothesized that Tween 80 could change the nutrient availability by changing the size of nutrient particles in media. We therefore measured the particle size distribution of TSB with and without Tween 80. Addition of 0.1% Tween 80 reduced the mean particle size and the polydispersity index (**Table 2**), indicating that the particles were not only smaller, but also more homogeneous in size.

**Table 2:** The particle size distribution in Tryptic Soy Broth (TSB) with or without 0.1% Tween 80.

	*Z*-average [nm]	PdI
TSB	1515 (±171)	0.754
TSB with 0.1% Tween 80	100 (±61)	0.252

*Sizes are given as the Z-averages (d.nm), and standard deviations are shown in brackets (n = 3). The polydispersity index (PdI) is also given.*

### Tween 80 Permeabilized a Model Membrane

**FIGURE 6:**
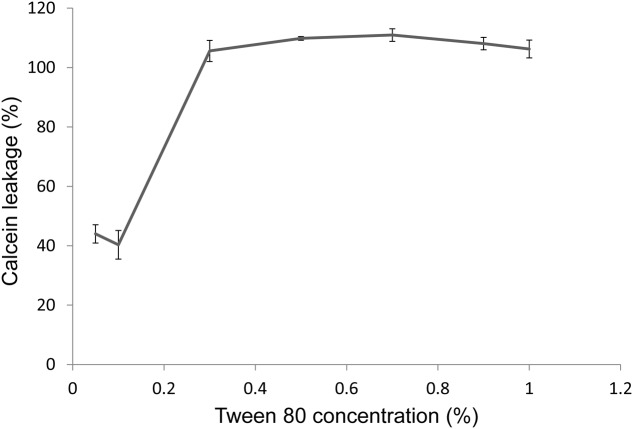
**Permeabilization of a model membrane after 1 h treatment with Tween 80, measured by the leakage of calcein from vesicles suspended in TSB.** Leakage is measured as fluorescence intensity, as vesicles become increasingly fluorescent as the calcein concentration within decreases. Values are normalized to a control sample in which 100% permeabilization is achieved by addition of 0.5% (v/v) Triton X-100. Error bars show the standard deviation of the mean (*n* = 3).

Another way for Tween 80 to affect nutrient availability could be through membrane permeabilization. We will discuss this issue in more detail in the discussion section. To test this hypothesis, we produced calcein-loaded membrane vesicles consisting of 50% cardiolipin and 50% phosphatidylglycerol. Tween 80 permeabilized the model membrane in a concentration dependent manner (**Figure 6**). The concentration used in bacterial assays, 0.1% Tween 80, resulted in 40% release of calcium from the vesicles after 1 h incubation.

## Discussion

Tween 80 is a commonly used emulsifier both in the laboratory and in industry. It is often included in laboratory experiments without any comments or evaluations of possible side effects. In this study, we show that Tween 80 affects microbial growth either positively or negatively. These effects cannot be ignored, and the use of Tween 80 should be reconsidered.

We hypothesized that Tween 80 affects both planktonic and biofilm growth, and we tested our hypothesis on three different bacteria. Tween 80 stimulated the growth of *S. aureus* both in batch culture and as biofilms. Several other studies have investigated the effect of Tween 80 on biofilm formation. Toutain-Kidd et al. (2009) have shown that Tween 80 can inhibit biofilm formation in *P. aeruginosa* and *S. aureus.* For *S. aureus*, 8 of the 12 clinical isolates tested were inhibited by 0.01% Tween 80. All the isolates that were not inhibited showed high levels of lipase activity, which could result in cleavage of Tween 80 at its ester bond, producing oleic acid, and polyethylene (Banin et al., 2006) sorbitan. However, the authors seem to have only examined addition of Tween 80 from the beginning of biofilm formation, though it is not clearly stated in the article. We observed similar trends for addition of Tween 80 at the onset of biofilm formation, which also suggests that lipase activity did not affect the results in our study. However, we also show that Tween 80 can stimulate biofilm growth for *S. aureus* but not for *L. monocytogenes* and *P. fluorescens* when added to mature biofilms.

We suggest that Tween 80 can affect planktonic bacteria and biofilms through multiple mechanisms. The surfactant may affect nutrient availability in three ways: (i) by reducing the size of nutrient particles, which indicates dispersal of, e.g., peptide aggregates and a higher surface to volume ratio, (ii) by acting as a nutrient source itself (Partanen et al., 2001), and (iii) by increasing the permeability of membranes. Above a critical concentration, this would be detrimental to the cell but below the critical concentration it might have a growth-promoting effect by slightly stressing the cell and possibly allowing for higher nutritional uptake from the media (Taoka et al., 2011). Application of electric fields can be utilized to create membrane permeabilization. It has been shown that moderate electric fields can result in reduced lag time of *Lactobacillus acidophilus* (Loghavi et al., 2008, 2009). Hence, some degree of permeabilization can be growth promoting. We show that Tween 80 can permeabilize a model membrane, and although this system is much simpler than a real bacterial membrane, the result demonstrates a potential for membrane perturbation. The permeabilizing properties of Tween 80 will depend on the specific lipid composition of the membrane (both inner and outer for Gram-negative bacteria), on membrane proteins, and on the properties of the peptidoglycan layer and surrounding extracellular polymeric substances. Therefore, this effect will be strain dependent and may explain the different effects seen for different strains in this study.

Tween 80 may also affect bacterial growth through its properties as a ionophore, reversibly binding (and hence transporting) metal ions such as K^+^, Na^+,^ and Ca^2+^ (Thoman, 1986). Ions take part in many cell processes, such as signaling and enzyme activity. Potassium has recently been shown to mediate electrical signals within *Bacillus subtilis* biofilms, which in turn was correlated with coordinating metabolism (Prindle et al., 2015). Divalent cations, such as magnesium, calcium, and iron, are important for biofilm cohesiveness. If these ions are removed by chelating agents such as EDTA, the bacteria in the biofilm will detach and some will be killed (Banin et al., 2006). We can imagine that Tween 80 could transport and concentrate divalent cations in the biofilm matrix. Finally, Tween 80 has been shown to interact with lipases (Liu et al., 2000) and proteins (Wang et al., 2008), which could have profound effects if an essential lipase or protein was activated, transported, or isolated by Tween 80. The mechanism by which Tween 80 affects a specific microorganism is probably complex and could include elements of all of the features mentioned above.

We found that oleic acid was present in the Tween 80. Oleic acid has been shown to inhibit biofilm formation in *S. aureus* when added in the initial adhesion step, while it stimulates biofilm formation when added after primary adhesion. (Stenz et al., 2008) This is exactly the effect we observed on *S. aureus*, and we therefore included treatments with a mix of 0.1% Tween 80 and 0.1% oleic acid in our study. However, we observed no further increase in biomass of *S. aureus* when oleic acid was added together with Tween 80 (**Figure 3B**). The oleic acid moiety present in Tween 80 may be responsible for the observed effects in *S. aureus*. Addition of oleic acid increased the biomass of *L. monocytogenes* and *P. fluorescens*, whereas Tween 80 alone did not (**Figure 3**).

In addition to the direct effects on planktonic and biofilm growth, we show that Tween 80 lowered the antibacterial efficacy of hydrophobic antimicrobials against biofilms. Rifampicin can be produced by *Amycolatopsis rifamycinica* (Bala et al., 2004) and works by inhibiting RNA synthesis in bacteria (Hartmann et al., 1967). Isoeugenol is an essential oil that can be isolated from plants and it works by permeabilizing the bacterial membrane (Hyldgaard et al., 2015). Others have also observed that addition of Tween 80 has an antagonistic effect on the activity of antibacterial compounds. Juven et al. (1994) performed a study where they tested the antibacterial efficacies of thyme oil or thymol against *Salmonella typhimurium* and *S. aureus.* The authors observed that addition of increasing amounts of Tween 80 resulted in reduced antibacterial efficacy of the essential oils. The researchers suggested that the observed effects could be explained by Tween 80’s ability to solubilize more thymol in the water phase and hence decrease the concentration of thymol in the bacterial membrane. It was hypothesized that thymol acts on bacterial proteins, and another suggestion was that Tween 80 could reduce the binding between thymol and bacterial proteins by making them both more hydrophilic. Krsta et al. (2014) investigated the effect of addition of Tween 80 to triclosan, platensimycin, and linezolid against *Streptococcus agalactiae* and Methicillin-Resistant *S. aureus*. For *S. agalactiae*, addition of Tween 80 abolished the antibacterial effects of triclosan and platensimycin in concentrations as low as 0.02% but showed no effects on linezolid in the concentration range 0.02–0.1%. For *S. aureus*, addition of Tween 80 reduced the antibacterial effects of triclosan, increased the antibacterial effects of platensimycin and showed no effect on linezolid. It was hypothesized that Tween 80 could circumvent antibiotics acting on the FASII pathway (a cyclical pathway for fatty acid synthesis in bacteria) by providing the naturally occurring fatty acid, oleic acid.

As Tween 80 is a commonly used emulsifier both in research and industries, the effects shown in this study call for re-evaluation of its use. It should be used with care in bacterial assays since it has strain-specific effects on planktonic and biofilm growth. The greatest effect was seen for the lowest concentration used in this study, 0.1%. The effects might be even greater at lower concentrations. This should be investigated further in the future. In this study, the emulsifier was shown to promote both planktonic and biofilm growth of *S. aureus*, whereas it inhibited *L. monocytogenes* and *P. fluorescens*. Addition of Tween 80 to disperse a compound of interest can therefore provide biased results due to the direct effect of Tween 80. Tween 80 is added to a wide range of foods, pharmaceuticals and cosmetics. A cream containing Tween 80 could affect the skin bacteria and foods and pharmaceuticals with Tween 80 could affect the gut microbiota. The knowledge obtained in this study stresses the need to reevaluate the use of Tween 80 and to consider its effects on different bacterial strains.

## Author Contributions

CN: Experimental design, experimental work, data analysis, and manuscript preparation. JK: Experimental design, data analysis, and manuscript preparation. TM: Experimental design, data analysis, and manuscript preparation. TS: Experimental design, data analysis, and manuscript preparation. RM: Experimental design, data analysis, and manuscript preparation.

## Conflict of Interest Statement

The authors declare a potential conflict of interest, as the work was jointly funded by the Graduate School of Science and Technology at Aarhus University and DuPont Nutrition Biosciences. However, the work does not promote Dupont’s products or discredit the products of their competitors.
